# Culturally Sensitive Health Education in the Caribbean Diaspora: A Scoping Review

**DOI:** 10.3390/ijerph18041476

**Published:** 2021-02-04

**Authors:** Mashtura Hasan, Harmandip Singh, Farzanna Haffizulla

**Affiliations:** 1 Dr. Kiran C. Patel College of Allopathic Medicine, Nova Southeastern University, Fort Lauderdale, FL 33314, USA; hs801@mynsu.nova.edu; 2 Department of Internal Medicine, Dr. Kiran C. Patel College of Osteopathic Medicine, Nova Southeastern University, Fort Lauderdale, FL 33314, USA

**Keywords:** Caribbean diaspora, health interventions, health education programs, diabetes, nutrition, physical activity, culturally sensitive, intersectionality, inclusion, health equity

## Abstract

Context: The Caribbean diaspora in the United States is a diverse community that is afflicted with high morbidity and mortality due to preventable chronic diseases. Objective: Our goal is to determine which culturally sensitive health and nutrition educational modalities have the highest efficacy for improving general health in the Caribbean diaspora. Methods: A scoping literature review was performed on the MEDLINE, CINAHL, and Web of Science databases using terms related to health and nutrition in the Caribbean population. Original, peer-reviewed research published from 2010 to 2020, which took place in the U.S. and Caribbean countries, were included in our review. Results: We identified a total of nine articles that met our inclusion criteria. Rate differences for individual education program features were calculated to assess the likelihood of a positive impact on diet, physical activity, and diabetes. Conclusion: Our review helps to identify key educational modalities targeting diabetes, diet, and physical activity levels that can be used to meet the health and nutritional needs of the Caribbean diaspora population.

## 1. Introduction

The goal of this scoping review is to highlight the most culturally sensitive and effective health educational modalities available for the Caribbean diaspora in the United States. Culturally sensitive interventions, defined in literature as processes that incorporate a specific population’s culture (e.g., norms, beliefs, values, and language), can be useful for addressing the needs of the Caribbean diaspora [1]. This scoping review is a derivative of the Caribbean Diaspora Healthy Nutrition Outreach Project (CDHNOP) research and community health initiative, which strives to improve health outcomes and reduce health disparities amongst Caribbean immigrants and their families in the US [2]. The Caribbean diaspora in the United States is a very diverse population made up of people originally from the Caribbean region. While these communities can be further subdivided by ethnic ancestry, we currently intend to treat them as a whole, with plans in the future for segmentation using the different subpopulations. As of 2017, approximately 4.4 million immigrants in the United States are from the Caribbean. Greater than 90% of these immigrants come from just five Caribbean territories: Cuba, Dominican Republic, Haiti, Jamaica, and Trinidad and Tobago. The three states with the largest concentration of Caribbean immigrants are Florida, New York, and New Jersey [3,4]. However, despite the significant population size in the United States, the Caribbean diaspora is significantly underrepresented with respect to focused research and health policy implementation. The CDHNOP’s intercept survey research results have indicated that as much as 92% of participants reported that these revised education materials were helpful in making positive food and beverage choices. These surveys were performed to assess the effectiveness of the Caribbean-focus-group-generated culturally tailored education materials. In accordance with our aims, implementation and assessment of a community-based health behavioral modification program would be an appropriate next phase of the CDHNOP and could improve the overall health of the community, reduce the burden on the healthcare system, and eventually be applied to the Caribbean population in other regions [2]. 

Although there are racial and ethnic health disparities, there is a lack of accurate data categorizing risks specific to the Caribbean diaspora. This is due to the paucity of studies in addition to the classification of Caribbean immigrants under the broad racial category of “Black” or “Other” in national data [5,6,7]. This issue is further complicated by the complex racial and ethnic heterogeneity of the Caribbean immigrant population. The Caribbean diaspora consists of multiple subgroups that are identified by their own specific ethnic ancestry, most notably that of Afro-Caribbeans and Indo-Caribbeans [8]. For example, Caribbean-born Blacks have been shown to have better health outcomes than US-born Blacks [6]. Overall, the rates of cerebrovascular diseases remain higher among African origin populations in the US. However, the few studies that specify subgroups have highlighted differences in disease and risk factors between the Caribbean population and other ethnic groups, including African Americans [9,10]. One study found higher stroke mortality in the Caribbean population compared to other ethnic groups. Cardiovascular mortality in the Caribbean population was also higher compared to other groups, similar to the risk in African Americans [9]. Considering rates of hypertension, a systematic review by Bidelescu et al. reported a higher prevalence among Caribbean blacks compared to that of West African blacks and Caucasians [11]. Additionally, despite the lower obesity rates among US-born blacks, both Africans and Caribbeans living in the same US geographic area share similar high odds of diabetes. Research has consistently shown health disparities in the US Caribbean population; however, the differences in risk profiles demonstrate the need for more specific epidemiological and population health data [10]. In addition, health education and lifestyle interventions tailored to be culturally appropriate can have a positive impact on the burden of preventable diseases in the Caribbean population.

Previously, culturally appropriate health interventions have been studied for different minority populations. In particular, there has been a surge in research on health interventions for African American populations in order to prevent disease and reduce health inequalities due to ethnicity. These focused health interventions are important for minority populations because much of the evidence in prior health intervention studies has its roots in populations of European origin from developed nations [12,13]. When factoring in psychosocial support for African American healthcare issues, studies have described faith-related, community-related, empowerment-related, and culturally appropriate intervention strategies [14]. A systematic literature review in 2015 by Smalls et al. assessed that community-based interventions generally led to significant reductions in glycosylated hemoglobin (A1c) levels in African Americans with type 2 diabetes [15]. Utz et al. described a diabetes self-management approach that revolved around group story-telling and hands-on activities alongside individual goal-setting sessions that showed some improvements in glycosylated hemoglobin (A1c) levels and self-care activities [16]. This was further explored in a 2014 study with a 2-year follow-up in a new set of 25 African American adults, showing persistence in improved outcomes in A1c levels and self-management skills [17]. Peña-Purcell et al. implemented an empowerment-based diabetes self-management program that was culturally adapted for rural African American communities that showed significantly increased levels of diabetes knowledge, self-care behaviors, and health status [18]. Research has also shown that mental health is a very important element in managing chronic health conditions and improving health outcomes for older African Americans [19]. Additional culturally appropriate approaches have been effective for smoking cessation in African Americans as well, such as narrative communication and targeted digital videos [20,21]. Overall, these studies suggest that a tailored approach for health interventions may have a more significant impact on health outcomes in minority populations instead of a one-size-fits-all approach.

Interventions have shown to be effective tools in disease prevention and management. The US Preventative Services Task Force concluded that behavioral counseling interventions that promote healthy diets and physical activity have had some positive net benefit in adults with cardiovascular risk factors [22]. The high incidence rate of disease in the Caribbean population is especially concerning, considering the low rate of screening and regular check-ups in the population [23]. Multiple factors such as socioeconomic variables, cultural beliefs, language barriers, and structural barriers contribute to these statistics [24]. Despite increasing numbers of Caribbean immigrants, there have been few studies on effective and culturally appropriate interventions tailored for the Caribbean population in the US. Few studies have evaluated educational techniques to improve health outcomes that are specifically tailored for the Caribbean diaspora. The objective of this scoping literature review is to assess effective health educational modalities that can be appropriately adapted and applied to improve health outcomes in the Caribbean diaspora. In order to address this, we generated the following research question: In the Caribbean diaspora, what educational health programs are available to improve dietary or physical activity outcomes?

## 2. Materials and Methods

### 2.1. Search Strategy

Our team adopted and modified the Preferred Reporting Items for Systematic Reviews and Meta-Analyses Extension for Scoping Reviews (PRISMA-ScR) protocol for performing our scoping literature review [25]. We used PICO search terms that evaluated Caribbean diaspora populations, the type of education program done as an intervention, and the outcomes measured. The specific search terms that were used in our search for each PICO category are shown in Table 1. The optional “comparison” component of the model was excluded from the search expression to avoid removing relevant literature from the results. The boolean operators “and” and “or” were used across and within parameters, respectively. The studies included were peer-reviewed, written in English, and published within the last ten years (2010–2020). The databases MEDLINE (264), CINAHL Complete (35), and Web of Science (120) were used to find publications. The types of studies included were journal articles, reviews, systematic reviews, meta-analyses, observational studies, and randomized controlled trials. 

Web search history was also recorded in order to investigate the availability and accessibility of information related to the Caribbean diaspora. The information recorded included the web search terms and the corresponding number of articles that were subjectively identified to be related and queried in the literature review. Every paper that was identified in our search was bookmarked, saved, and then added to the literature review extraction chart. The chart detailed the title, year, author(s), methods, key findings, theme, and sources found. The sources were also added to EndNote for citations.

### 2.2. Inclusion Criteria

**Figure 1:**
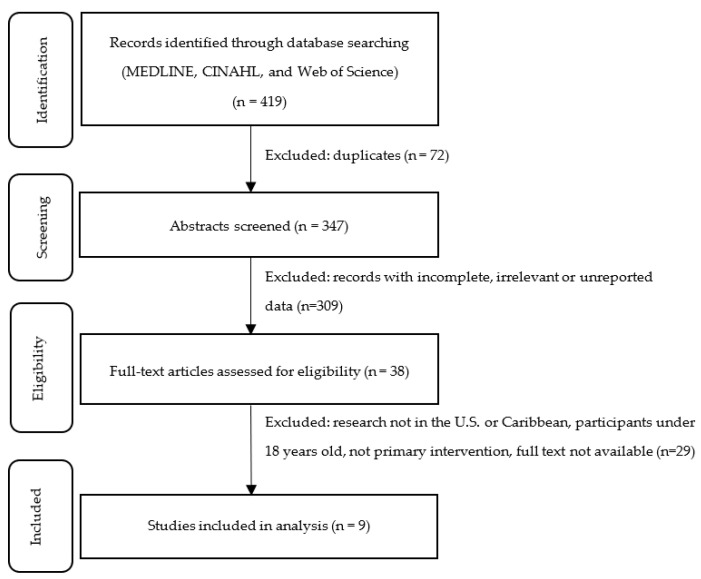
PRISMA-ScR (Preferred Reporting Items for Systematic Reviews and Meta-Analyses Extension for Scoping Reviews) flow diagram of identification, screening, eligibility, and inclusion.

A total of 419 papers from the 3 listed databases were identified from our search strategy performed on 12 March 2020. After the removal of duplications, 347 unique papers remained. Two independent investigators conducted an initial review of the abstracts associated with each published work to ensure each was appropriate to the topic of Caribbean diaspora diet or physical activity education programs. Studies were also excluded if it was not an original peer-reviewed research report or had insufficient information on the educational program conducted or outcomes measured. The remaining 38 papers were read and evaluated in their entirety. Papers were further limited to research undertaken in the United States and Caribbean countries and those that studied adult populations (defined as age >18 years old). These parameters were implemented in order to focus on research that could be applied to the growing Caribbean population in the US and address their comorbidities. A total of 9 papers were retained after full evaluation and included in our analysis. The selection process is illustrated in Figure 1.

### 2.3. Data Extraction

A data extraction chart was used to collect information on the nine papers that met our inclusion criteria. The two investigators independently filled out the data-charting form and discussed the results jointly. Studies were grouped into the themes of diet and physical activity. Many papers in our scoping review also assessed diabetes outcomes; therefore, it was included as an additional theme. The Template for Intervention Description and Replication (TIDieR) provides a 12-item checklist to improve the reporting and replicability of interventions [26]. We created a modified TIDieR table that was suited to the information we had available in our papers and incorporated additional items relevant to our scoping review. Our modified TIDieR table is presented in Table 2 and includes the additional items of theme, study design, quality assessment, and primary outcomes. To systematically analyze program features, each component of the educational program was extracted into our modified TIDieR table. We considered education programs to be culturally sensitive if they incorporated cultural or language tailoring, as referenced in the “Tailoring” column of Table 2.

### 2.4. Quality Assessment

Two investigators independently performed a quality assessment using the NIH quality assessment tool for the appropriate study type [36]. The investigators used the checklist criteria to assess the risk of bias of each study due to flaws in study design or implementation. The quality of each study was rated as “good”, “fair”, or “poor”. Discrepancies on ratings between the two reviewers were discussed and resolved. The final quality assessment rating can be found in our modified TIDieR table (Table 2). Additionally, the results of this NIH quality assessment tool can be found in the Appendix A (Table A1 and Table A2).

### 2.5. Data Analysis

We were unable to perform a meta-analysis due to a lack of homogeneity between education program types and the outcomes that were measured. Instead, we applied a previously defined method of calculating success rate differences that included and excluded the feature to determine which education program features are associated with successful outcomes [37,38]. Each study identified through the scoping review was evaluated on whether it had a program feature and whether the study documented a positive change in one or more outcome parameters based on our themes: diet, diabetes, and physical activity. The success rate difference of each feature on the outcome parameter was calculated from the difference between the success rate with the feature and the success rate without the feature. A success rate difference closer to 1.00 indicated a more positive association with the program feature and the outcome. Therefore, the more positive the success rate difference, the more likely it is that having the feature is associated with a positive effect on the outcome. A success rate difference of 0 suggests that the education program feature has no influence on the outcome. Negative success rate differences, closer to −1.00, indicate that not having the program feature was associated with a positive outcome.

## 3. Results

### 3.1. Description of Studies

Overall, nine studies [27,28,29,30,31,32,33,34,35] were analyzed in this scoping literature review. A summary of the study characteristics can be found in Table 2. Three interventions took place in the United States, and six were conducted in the following Caribbean countries: Jamaica (1), Dominican Republic (2), Trinidad and Tobago (2), Guadeloupe (1). Seven papers examined interventions on education or behavior modification to improve the nutritional status or glycemic control of the participants. The two remaining papers studied the efficacy of an exercise program in increasing physical activity levels in Caribbean populations. Diabetes was the most common comorbidity, which was assessed in five studies.

### 3.2. Analysis of Features

Success rate differences for each educational program feature are found in Table 3. We identified and compared 33 distinct program features within 9 broad categories. Of these 33 program features, 21 features appeared in fewer than 5 studies, which makes discerning their generalizability more difficult. The remaining 12 characteristics appeared in 5 or more studies. Features with a positive success rate difference are highlighted in yellow, and features with a negative success rate difference are highlighted in blue. The magnitude of the positive or negative association of the feature with the outcome is indicated by the integer value.

#### 3.2.1. Diet

Seven interventions [27,28,29,30,32,33,34] assessed outcomes related to diet, including confidence in healthy cooking, nutrition knowledge, diet adherence, and consumption of adequate nutrients. Four [27,32,33,34] of these interventions documented statistically significant positive diet outcomes following intervention implementation. Features with positive success rate differences include language or literacy tailoring, cultural tailoring, delivery by a research team or a multidisciplinary team, delivered online or by written literature, through interactive/discussion or feedback, and interventions that included diet and psychosocial content. Interventions that were less than six months, with less than two intervention sessions per month, were also moderately associated with positive diet outcomes. Delivery of the diet education through audio–visual means was associated with high success.

#### 3.2.2. Diabetes

Only one [32] out of the six [27,28,29,30,31,32] studies that assessed diabetes did not report a statistically significant decrease in HbA1c levels in intervention participants. Features with a positive success rate difference included programs designed for the individual or the healthcare provider, delivery by the healthcare provider, and, in a primary care or hospital-based setting, delivery face-to-face or through written literature or telephone, through didactic teaching or self-management education or feedback or diaries and reports. Programs that were six or more months were also associated with a positive likelihood of success. Programs that included diabetes content are associated with a positive success rate difference.

#### 3.2.3. Physical Activity

Six studies [27,28,29,30,34,35] assessed physical activity as an outcome through increased physical activity levels, body mass index, and body weight. Two [34,35] of these studies reported statistically significant improved physical activity outcomes. Programs that occurred at a community setting, such as a fitness center, were moderately associated with having a positive impact on physical activity. Features that were highly associated with improved physical activity measures included interventions delivered by a multidisciplinary team and those that incorporated family support.

## 4. Discussion

There is limited research on effective health education strategies for the Caribbean population in the United States. Of the nine health education program studies our scoping literature review identified, three studies took place in the United States. As the Caribbean population in the United States continues to grow, more culturally appropriate education programs are required to meet their health needs and to prevent morbidity and mortality associated with diseases such as diabetes, cancer, stroke, and heart disease.

This review identified three themes of health education programs: diabetes, diet, and physical activity. Identification of successful program features within these categories has the potential to decrease comorbidities and chronic health conditions in the Caribbean diaspora. In 2018, the CDC estimated that 34.1 million adults, or 13.0% of the US adult population, had diabetes. Additionally, non-Hispanic Blacks had the highest incidence of diabetes. Projection trends show that the diabetes prevalence has steadily increased since 1999 and will continue to grow [39]. Previous research has shown further racial disparities with aggregated health data. For example, Afro-Caribbeans have a higher prevalence of diabetes and stroke compared to other African ethnic groups and Caucasians [5]. Risk factors for diabetes include smoking, overweight and obesity, physical inactivity, high blood pressure, and high cholesterol. Weight management, physical activity, and routine diabetes care can prevent diabetes-related complications [39].

Our scoping review results offer a bright outlook for using culturally tailored health education in the Caribbean diaspora. Our findings suggest that certain program features have a dramatic impact on patient health outcomes. Programs that are delivered in a clinical environment by a healthcare professional and center on patient self-management education are associated with a significant positive success rate with respect to patient HbA1c levels. This finding is meaningful and correlates with data from the parent CDHNOP study that showed most of the Caribbean focus group participants obtained health information from their doctor’s office(s) [2]. In contrast, features such as interventions focused on behavior-related tasks and delivery of the intervention at a participant’s home or by a nonhealthcare professional are shown to have less influence on patient HbA1c levels. Factors that may have some impact on these features include lack of access to healthcare and public assistance, lack of insurance, and various psychosocial issues [24]. These findings bear some similarities to previous systematic reviews performed on socially disadvantaged populations and women of black African, Caribbean, or Hispanic ethnicity [37,38]. Glazier et al. [37] found that cultural tailoring of interventions, community educators leading the education program, individualized one-on-one programs, inclusion of treatment algorithms, “focusing on behavior-related tasks”, feedback, and high-intensity health education provided over a long duration provided the most reliable positive effects on improving diabetes care in socially disadvantaged populations. Gucciardi et al. [38] examined positive success rates of intervention features across multiple outcomes related to improving diabetes education, including diet, anthropometric and physical activity, and HbA1c. Their findings indicated that hospital-based education, group programs, usage of situational problem-solving, frequent sessions, and using dietitians as program providers had the broadest positive rate differences, affecting at least three outcomes. Program features that had a positive success rate difference closer to 1.00 included program delivery within participant communities or homes, interactive group discussions, specific feedback provided to participants by health education providers, diaries or log keeping, incorporation of psychosocial aspects, supervised exercise, and tailoring program features in the participant’s native language.

Nutrition knowledge and confidence in healthy food preparation were additional outcomes evaluated in our scoping review. Our results show that the Caribbean diaspora may benefit from health education programs that specifically focus on cultural- and language-relevant nutrition education videos and visuals. Similar to the CDHNOP study, several culturally oriented studies in the past have noted similar effects. Attridge et al. [40] found that culturally sensitive health education in ethnic minorities had a positive impact on glycemic control and patient knowledge of diabetes, along with the associated healthy lifestyles. Israel et al. [41], Masuda et al. [42], and Naqshbandi et al. [43] each noted unique benefits from using community-based participatory research to minimize health disparities. These initiatives are valuable because they build collaborative partnerships between multiple stakeholders, emphasize culture and ethics, and link community action with social change.

Our findings also suggest physical activity programs that involved family support and a multidisciplinary approach in a community setting were associated with increased exercise levels and weight loss in participants. Programs incorporating physical activity in the Caribbean diaspora population will be necessary to decrease the prevalence of obesity and its associated negative health outcomes. Structural, societal, and cultural barriers impact the ability of individuals to increase their level of physical activity [24]. Alvarado et al. [24] found that Afro-Caribbean women in Barbados had a perceived reduction in their access to convenient and affordable exercise programs. The associated costs for programs incorporating physical activity were a common limitation noted by the reviewed articles [34,35]. In one study, 92 percent of participants reported that cost was an obstacle in continuing their fitness center membership, demonstrating a financial limitation in promoting sustainable behavioral modification [34]. Antoine-Jonville et al. [35] provided a free 10-week program to study participants and also recognized that future interventions should aim to decrease the costs of exercise programs. In addition to resource limitations, the Afro-Caribbean women interviewed by Alvarado et al. also referenced differing health beliefs about chronic disease and the role of physical activity in weight loss. For example, some of the women perceived chronic disease as random and inevitable and were, therefore, not convinced of the association between an active lifestyle and long-term health [24]. These findings highlight the importance of addressing cultural, societal, and cost limitations when constructing an appropriate physical activity program for the Caribbean diaspora.

## 5. Limitations

This scoping review has several limitations. First, only nine primary education program articles were found through our literature review, limiting the data available for analysis of effective education features. Due to the specific search performed, it is possible that other health education studies in this population were excluded. Second, the lack of homogeneity of studies and variable outcome parameters prohibited us from performing a meta-analysis. Additionally, while we hope to utilize this information in the Caribbean diaspora population, we included health programs that took place in Caribbean countries in our analysis due to the limited number of studies that were conducted in the United States. Our data may not fully account for different effect modifiers and confounding factors that are present in this other region. We were also limited to the descriptions of the educational program, as detailed in the original paper. This posed another challenge during data extraction as some papers did not describe study characteristics in the detail necessary for our modified TIDieR table.

## 6. Conclusions

There is a lack of data behind effective and culturally sensitive health education programs tailored for the Caribbean population in the United States. This scoping literature review highlights several health education program features that are associated with high success rates in improving diabetes, nutrition, and physical activity in the Caribbean population. Implementing effective health education in the Caribbean diaspora can improve the health of each individual member, reduce the burden on the healthcare system, and will eventually be applied to this underserved, underrepresented demographic nationally. 

## Figures and Tables

**Table 1:** PICO search terms used in the search strategy.

	Search Terms
Population	Caribbean or West Indies or Afro-Caribbean
Intervention	education or coaching or video or printed materials ortraining or radio
Outcomes	nutrition or diet or exercise or physical activity

**Table 2:** Modified TIDieR (Template for Intervention Description and Replication).

Name	Theme	Objective	Study Design	Program	Program Provider	Delivery
Culturally tailored intervention for Puerto Ricans with type 2 diabetes [27]	Diabetes Diet Physical activity	To determine the effect of an Information–Motivation–Behavioral Skills (IMB) intervention on diabetes self-care behaviors and glycemic control.	Randomized controlled trial (RCT)	The information–motivation–behavioral skills (IMB) model of health behavior change informed the design of a brief, culturally tailored diabetes self-care intervention for Puerto Ricans with type 2 diabetes. A flipchart, available in English and Spanish, presented information on food label reading, diet adherence, physical activity, and glycemic control (HbA1c). The session consisted of a brief introduction, information on diabetes prevalence in Puerto Ricans, motivational interviewing, and behavioral skills building. Patients were also provided with supplemental materials.	Bilingual medical assistant of Puerto Rican heritage	Group
Community-based intervention for type 2 diabetes control [28]	Diabetes Diet Physical activity	To evaluate the effectiveness of lay diabetes facilitators (LDFs) to increase knowledge and improve control among persons with diabetes.	Nonrandomized controlled trial	Two types of education sessions were conducted: one-to-one and group. The group sessions were conducted at the health centers on days when clients came for regular three-monthly visits and were limited to 10–12 patients. Those unable to attend had education sessions at home when the LDFs visited. The education gave focus to the timing of meals in relation to prescription of diabetes medication, physical activities, blood glucose monitoring, and hypoglycemia.	Community persons trained as lay diabetes facilitators	Individual and group
Applying the Stages of Change model to type 2 diabetes care [29]	Diabetes Diet Physical activity	To improve glycemic control among Type-2 diabetics using patient-physician consultations guided by the Stages of Change (SOC) model.	RCT	The intervention consisted of identifying each patient’s SOC for managing their diabetes by diet, exercise, and medications and applying personalized, stage-specific care during the patient–physician consultations based on the SOC model.	Physicians	Individual
**Name**	**Theme**	**Objective**	**Study Design**	**Program**	**Program Provider**	**Delivery**
Community-based lifestyle intervention program on type 2 diabetes and cardiovascular risk [30]	Diabetes Diet Physical activity	To assess the development of a cost-effective and sustainable approach to lifestyle modification in underdeveloped countries that can be implemented using community members as healthcare champions.	One group, pretest–posttest design	In a 1-day session, trained lifestyle educators from the USA trained ten lay community members to lead groups oriented to a lifestyle change. Community leaders met with assigned patient groups monthly for 1 year.	Lay community leaders	Group
Self-monitoring of blood glucose [31]	Diabetes	To assess the difference in glycemic control and coronary heart disease (CHD) risk levels of experimental type 2 diabetes patients provided with facilities for self-monitoring blood glucose and their counterparts without such facilities.	RCT	Intervention group was given glucose meters and testing strips that could last 3 months (90 days). Furthermore, members of the intervention group (glucose meters users) were trained on how to use the glucose meters and data documentation	Clinic staff	Group
Cooking for Diabetes Prevention and Management [32]	Diabetes Diet	To assess health behaviors of Caribbean–American women and to assess the usefulness of a website in increasing awareness of diabetes and diabetes prevention.	One group, pretest–posttest design	Online survey with seven 2-min videos of cooking instructions for modified traditional Caribbean meals	Research Fellows produced videos of the traditional Caribbean meals	Individual
Community-partnered nutrition intervention in rural communities [33]	Diet	To assess the feasibility and acceptability of culturally appropriate nutritional intervention programs in rural Dominican Republic	One group, pretest–posttest design	The intervention used a psychoeducational approach to develop a collaborative partnership between two not-for-profit organizations. The program consisted of (1) individualized nutritional counseling; (2) a brief culturally appropriate diet video in Spanish or Creole; (3) an interactive food card activity to enhance the skills learned in the video.	Members of the Light a Candle Foundation	Individual
Curves Weight Management Program [34]	Diet Physical activity	To examine the effects and feasibility of the commercial Curves weight loss program among Hispanic, African American, and Afro-Caribbean breast cancer survivors	RCT	Participants were given access to Curves fitness centers and a Curves diet plan, which was taught by Curves staff via a standardized nutrition course that made use of a book, DVDs, and an instructor’s manual, all published by Curves.	Curves Weight Management Program staff and bilingual nutrition instructors	Group and Individual
Swimming-based physical activity promotion program [35]	Physical activity	To promote short- and long-term participation in physical activities through the developmentof personal skills, self-confidence, and social networking	One group, pretest–posttest design	Each participant followed the same schedule during the intervention period: two swimming sessions (1 h each) in the first week and one swimming session plus another 2.5-h session of sailing or kayaking in the following week, with this alternating pattern repeated for a total of five times.	Qualified sports educator	Group
**Name**	**Setting**	**Duration/Frequency**	**Tailoring**	**Adherence**	**Quality Assessment**	**Primary Outcomes**
Culturally tailored intervention for Puerto Ricans with type 2 diabetes [27]	Primary care clinic at an urban hospital in the Northeast US	Single 90 min (60 min allocated to diet content and 30 min to exercise content)	Language Culture Needs Assessment	Diet and Physical Activity adherence was measured with the Summary of Diabetes Self-Care Activities questionnaire (SDSCA).	Good	Laboratory blood testing showed mean HbA1c score decreased in both groups, but only the intervention group showed significant improvement from baseline (M = 7.76, SD = 1.37) to follow-up (M = 7.28, SD = 1.29), (*p* < 0.008). The control group’s HbA1c decrease from baseline (M = 7.45, SD = 1.58) to follow-up (M = 7.18, SD = 1.54) and was not significant, with a Bonferroni adjustment of 0.01 (*p* < 0.047).
**Name**	**Setting**	**Duration/Frequency**	**Tailoring**	**Adherence**	**Quality Assessment**	**Primary Outcomes**
Community-based intervention for type 2 diabetes control [28]	16 LDF health centers in Jamaica	Three monthly visits for a six-month period	Language Culture	LDFs used three patient self-monitoring forms (personal eating tracker, physical activity log, self-blood glucose readings) to track individual progress.	Fair	Laboratory blood testing showed that mean HbA1c for the intervention and comparison groups were similar at baseline (7.9% versus 8.0%; *p*-value >0.58). However, at 6 months, the intervention group had a reduction in HbA1c of 0.6%, while the comparison group had an increase of 0.6% (*p* < 0.001).
Applying the Stages of Change model to type 2 diabetes care [29]	Ste. Madeleine Health Centre (SMHC) in south Trinidad	Two days per month for 48 weeks	Needs Assessment	58 of the 61 patients completed the intervention	Good	Laboratory blood testing showed the mean increase in HbA1c for the intervention group was 0.52% (SE 0.17) compared to that at baseline. The change in glycemic control for the control group was a mean increase in HbA1c of 1.09% (SE 0.18) compared to that at baseline.
Community-based lifestyle intervention program on type 2 diabetes and cardiovascular risk [30]	Club organization in Villa Juana, Santo Domingo, Dominican Republic	Once a month for one year	Language Needs Assessment	Of the 79 patients enrolled in group therapy during the first 6 months, 10 attended all five meetings, 36 attended four, 19 attended three, and 14 attended two; 59 patients returned for follow-up HbA1c measurement.	Good	Patients showed significant improvements after 6 months in systolic blood pressure (*p* = 0.001), diastolic blood pressure (*p* = 0.000002), and HbA1c (*p* = 0.015). HbA1c improved further at 1 year (*p* = 0.005).
Self-monitoring of blood glucose [31]	Lifestyle Disease Clinics (primary care clinic) in Tobago	One training session with a six-month follow-up period	Language	Weekly follow-up phone call for six months	Fair	Laboratory blood testing showed that mean HbA1c of the intervention group improved significantly at three months (9.6 ± 0.3% vs. 7.8 ± 0.3%, *p* <0.001) and six months (9.6 ± 0.4% vs. 7.5 ± 0.3%, *p* < 0.001); CHD risk level of the intervention group was reduced by nearly one-half after three months (7.4 ± 1.3% vs. 4.3 ± 0.7%, *p* = 0.056).
**Name**	**Setting**	**Duration/Frequency**	**Tailoring**	**Adherence**	**Quality** Assessment	**Primary Outcomes**
Cooking for Diabetes Prevention and Management [32]	Online	One-time online survey	Language Culture	Not reported	Fair	Self-reported survey results showed that self-efficacy for cooking healthy before exploring the website was a mean of 3.52 between 40% and 60% confident (SD = 1.509) versus the after mean of 4.59 (closest to 80% confident or a “good” level of confidence, SD = 1.154; t = −10.353, df = 147; *p* < 0.001).
Community-partnered nutrition intervention targeting rural migrant communities [33]	Mobile medical clinics in 6 rural Dominican Republic	The one-session intervention was provided at three time-points. Participants were allowed to repeat the intervention, if desired.	Language Culture Needs Assessment	Not reported	Fair	Self-reported survey results showed that the difference between pre- and posttest scores was found to be significant for participants who had received the intervention for the first time: t(306) = 34.3, *p* < 0.001, for knowledge score; t(306) = 19.77, *p* < 0.001, for energy category; t(306) = 23.84, *p* < 0.001, for protection from illness category; t(306) = 28.70, *p* < 0.001, for growth category.
Curves Weight Management Program [34]	Curves fitness centers in New York City; Nutrition course took place at Columbia University Medical Center	Target goal to exercise 3 days/week for six months; nutrition course consisted of six 1-h weekly group sessions	Language Needs Assessment	Adherence to the exercise program was monitored using computerized Curves attendance logs. Attendance was recorded at the nutrition education sessions, and telephone make-up sessions were tracked.	Good	Anthropometric measurements showed that the intervention group lost an average of 3.3% (±3.5%) of total body weight at 6 months, which corresponds to an absolute weight loss of 2.9 (±3.1) kg. The control group lost an average of 1.8% (±2.9%) of body weight, which corresponds to an absolute weight loss of 1.4 (±2.5) kg. There were no changes in metabolic biomarkers using intent-to-treat analysis, as assessed by serum testing.
Swimming-based physical activity promotion program [35]	12 practice sites in Guadeloupe	2 sessions per week for 10 weeks	Language Culture Needs Assessment	Not reported	Fair	Self-reported quality of life survey showed no significant change (*p* > 0.05) for all domains, including physical health, psychological health, social relationships, and environment. A self-reported physical activity survey showed significant improvement in physical activity (*p* < 0.001).

**Table 3:** Success rate differences of education program features.

Program Feature	Diet	Diabetes	Physical Activity
Tailoring of the program			
Language or literacy tailoring: the program is tailored to the specific populations’ language or literacy level	0.50	−0.50	0.25
Cultural tailoring: the program is tailored to the specific populations’ culture *	0.35	−0.35	−0.05
Needs assessment: the program assesses each participant’s individual needs formally for the design of the content	0.17	−0.17	0.33
Program provider			
Health care provider: delivered by a licensed health care provider *	−0.17	0.67	−0.33
Community-educator-led: delivered by trained nonhealthcare personnel *	−0.35	−0.10	0.05
Multidisciplinary team: delivered by two or more types of program providers *	0.63	−0.63	0.88
Research staff: delivered by a trained research team *	0.63	−0.63	−0.25
Setting of the program			
Primary care: delivered in the primary care system (e.g., family practice) *	−0.17	0.67	−0.33
Community-based: delivered outside of the healthcare setting (e.g., local church) *	0.10	−0.55	0.50
Hospital-based: delivered in a clinic affiliated with a hospital *	−0.50	0.50	−0.25
Home: delivered at the residence of the subject *	0.07	−0.07	−0.29
Mode of program delivery			
Individual: delivered individually, one-on-one	−0.10	0.10	−0.05
Group: delivered to a group of participants	0.17	−0.17	0.33
Format of program			
Face-to-face: delivered face-to-face with participants	−0.63	0.63	0.25
Online: delivered through the internet *	0.63	−0.63	−0.25
Written Literature: delivered through written material (e.g., handbook) *	0.63	0.50	−0.25
Telephone: delivered by phone *	−0.50	0.50	−0.25
Audio–Visual: delivered through educational videos *	0.83	−0.33	−0.33
Method/type of instruction			
Interactive/discussion: education provided was delivered mainly by an interactive format *	0.55	−0.10	0.05
Didactic teaching: education provided was delivered mainly by lectures	−0.10	0.55	−0.05
Behavioral: focuses on behavior-related tasks (e.g., exercise, diet) using behavior-based programs	−0.07	0.07	0.29
Self-management education: comprehensive education that focuses on developing diabetes knowledge and patients’ ability to self-manage their diabetes *	0.07	0.57	−0.29
Family support: encourages family members to get involved in attending the session *	−0.50	−0.63	0.88
Feedback: interventionists provide specific feedback for participants to aid in monitoring aspects of their own management (e.g., diet and exercise feedback) *	0.63	0.50	−0.25
Diaries and Reports: a specific type of feedback activity, where food diaries, physical activity logs, and self-management of blood glucose logs were used by participants to record specific components *	−0.57	0.57	−0.29
Duration of the program			
Short Duration: length of the program is less than 6 months *	0.55	−0.55	0.05
Long Duration: length of the program is equal to or more than 6 months	−0.55	0.55	−0.05
Intensity of the program			
Low Intensity: less than 2 sessions per month *	0.55	−0.10	−0.40
High Intensity: equal to or more than 2 sessions per month	−0.55	0.10	0.40
Content of the program			
Diet: the program teaches diet-related content	0.57	0.07	−0.36
Physical activity: the program teaches exercise-related content	−0.33	0.33	0.33
Psychosocial: the program teaches psychosocial-related content *	0.71	−0.07	−0.29
Diabetes education: program is aimed at patients’ diabetes knowledge for specific diabetes management topics	−0.33	0.83	−0.67

* Feature appears in less than five studies. Features with a positive success rate difference are highlighted in yellow, and features with a negative success rate difference are highlighted in blue. The magnitude of the positive or negative association of the feature with the outcome is indicated by the integer value. A success rate difference closer to 1.00 indicates a more positive association with the program feature and the outcome. Therefore, the more positive the success rate difference, the more likely it is that having the feature is associated with a positive effect on the outcome. A success rate difference of 0 suggests that the education program feature has no influence on the outcome. Negative success rate differences, closer to −1.00, indicate that not having the program feature is associated with a positive outcome.

## Data Availability

Not applicable.

## Appendix A

**Table A1:** Results of quality assessment of the controlled intervention studies.

Year	Author	Q1	Q2	Q3	Q4	Q5	Q6	Q7	Q8	Q9	Q10	Q11	Q12	Q13	Q14	Quality
2009	Less	No	NA	NR	No	No	Yes	Yes	Yes	Yes	NR	Yes	Yes	Yes	Yes	Fair
2010	Osborn	Yes	Yes	Yes	Yes	Yes	Yes	No	Yes	Yes	NR	Yes	NR	Yes	Yes	Good
2011	Partapsingh	Yes	Yes	Yes	No	No	Yes	Yes	Yes	Yes	NR	Yes	Yes	Yes	Yes	Good
2011	Chidum	Yes	NR	Yes	No	NR	Yes	Yes	Yes	NR	NR	Yes	No	Yes	Yes	Fair
2013	Greenlee	Yes	Yes	Yes	NR	NR	Yes	Yes	Yes	Yes	NR	Yes	Yes	Yes	Yes	Good

Quality of included studies was assessed using the National Institutes of Health (NIH) Quality Assessment tool for Controlled Intervention Studies [27]. NR indicates “not reported”. NA indicates “not applicable”. Q1: Was the study described as randomized, a randomized trial, a randomized clinical trial, or an RCT? Q2: Was the method of randomization adequate (i.e., use of randomly generated assignment)? Q3: Was the treatment allocation concealed (so that assignments could not be predicted)? Q4: Were study participants and providers blinded to treatment group assignment? Q5: Were the people assessing the outcomes blinded to the participants’ group assignments? Q6: Were the groups similar at baseline on important characteristics that could affect outcomes (e.g., demographics, risk factors, comorbid conditions)? Q7: Was the overall drop-out rate from the study at the endpoint of 20% or lower than the number allocated to treatment? Q8: Was the differential drop-out rate (between treatment groups) at the endpoint of 15 percentage points or lower? Q9: Was there high adherence to the intervention protocols for each treatment group? Q10: Were other interventions avoided or similar in the groups (e.g., similar background treatments)? Q11: Were outcomes assessed using valid and reliable measures and implemented consistently across all study participants? Q12: Did the authors report that the sample size was sufficiently large to be able to detect a difference in the main outcome between groups with at least 80% power? Q13: Were outcomes reported or subgroups analyzed prespecified (i.e., identified before analyses were conducted)? Q14: Were all randomized participants analyzed in the group to which they were originally assigned, i.e., did they use an intention-to-treat analysis?

**Table A2:** Results of quality assessment of the pre–post studies with no control group.

Year	Author	Q1	Q2	Q3	Q4	Q5	Q6	Q7	Q8	Q9	Q10	Q11	Q12	Quality
2013	Antoine-Jonville	Yes	Yes	Yes	No	NR	Yes	Yes	NR	No	Yes	Yes	NA	Fair
2014	West-Pollak	Yes	Yes	Yes	NR	Yes	Yes	Yes	NR	No	Yes	Yes	NA	Good
2017	Maliszewski	Yes	No	Yes	Yes	Yes	Yes	No	NR	Yes	Yes	No	NA	Fair
2018	Thomas	Yes	Yes	Yes	Yes	NR	Yes	Yes	NR	Yes	Yes	No	NA	Fair

Quality of included studies was assessed using the National Institutes of Health (NIH) Quality Assessment tool for Before-After (Pre–Post) Studies with No Control Group [27]. NR indicates “not reported”. NA indicates “not applicable”. Q1: Was the study question or objective clearly stated? Q2: Were eligibility/selection criteria for the study population prespecified and clearly described? Q3: Were the participants in the study representative of those who would be eligible for the test/service/intervention in the general or clinical population of interest? Q4: Were all eligible participants that met the prespecified entry criteria enrolled? Q5: Was the sample size sufficiently large to provide confidence in the findings? Q6: Was the test/service/intervention clearly described and delivered consistently across the study population? Q7: Were the outcome measures prespecified, clearly defined, valid, reliable, and assessed consistently across all study participants? Q8: Were the people assessing the outcomes blinded to the participants’ exposures/interventions? Q9: Was the loss to follow-up after baseline 20% or less? Were those lost to follow-up accounted for in the analysis? Q10: Did the statistical methods examine changes in outcome measures from before to after the intervention? Were statistical tests done to provide *p*-values for the pre-to-post changes? Q11: Were outcome measures of interest taken multiple times before the intervention and multiple times after the intervention (i.e., did they use an interrupted time-series design)? Q12: If the intervention was conducted at a group level (e.g., a whole hospital, a community), did the statistical analysis take into account the use of individual-level data to determine effects at the group level?
